# Combined Therapy Planning, Real-Time Monitoring, and Low Intensity Focused Ultrasound Treatment Using a Diagnostic Imaging Array

**DOI:** 10.1109/TMI.2021.3140176

**Published:** 2022-06-01

**Authors:** Miles Thies, Michael L. Oelze

**Affiliations:** Department of Electrical and Computer Engineering, Beckman Institute for Advanced Science and Technology, University of Illinois at Urbana-Champaign, Urbana, IL 61820 USA

**Keywords:** Beam visualization, focused ultrasound, real-time, therapy monitoring

## Abstract

Low intensity focused ultrasound (FUS) therapies use low intensity focused ultrasound waves, typically in combination with microbubbles, to non-invasively induce a variety of therapeutic effects. FUS therapies require pre-therapy planning and real-time monitoring during treatment to ensure the FUS beam is correctly targeted to the desired tissue region. To facilitate more streamlined FUS treatments, we present a system for pre-therapy planning, real-time FUS beam visualization, and low intensity FUS treatment using a single diagnostic imaging array. Therapy planning was accomplished by manually segmenting a B-mode image captured by the imaging array and calculating a sonication pattern for the treatment based on the user-input region of interest. For real-time monitoring, the imaging array transmitted a visualization pulse which was focused to the same location as the FUS therapy beam and ultrasonic backscatter from this pulse was used to reconstruct the intensity field of the FUS beam. The therapy planning and beam monitoring techniques were demonstrated in a tissue-mimicking phantom and in a rat tumor *in vivo* while a mock FUS treatment was carried out. The FUS pulse from the imaging array was excited with an MI of 0.78, which suggests that the array could be used to administer select low intensity FUS treatments involving microbubble activation.

## I. Introduction

Focused ultrasound (FUS) is a versatile and powerful therapeutic tool. FUS allows for the non-invasive treatment of a wide variety of diseases by targeting FUS waves to a well-localized region [1], [2]. To ensure a safe and effective procedure, an FUS therapy requires therapy planning before treatment and real-time monitoring during treatment. Therapy planning is the process of aligning the FUS beam to the treatment region and determining a sonication pattern. Once treatment begins, real-time monitoring of the FUS beam’s position is important to make certain that the beam does not drift out of the treatment region due to tissue motion or movement of the ultrasound probe. An ideal FUS therapy system should provide the means for therapy planning, real-time monitoring, and the FUS treatment itself.

Traditionally, therapy planning is accomplished by capturing a number of ultrasound [3], magnetic resonance (MR) [4], or computed tomography (CT) images [5] of the area to be treated, which are then used to align the FUS source to the treatment region and determine a sonication pattern. This sonication pattern could be comprised of a single focal point, or if the treatment region is larger than the focal zone of the FUS source, then the sonication pattern could contain multiple focal points and mechanical scanning or electronic steering would be required to treat the entire region. Early studies on FUS therapy used single-element transducers or un-steered arrays and relied on mechanical translation to treat regions larger than a focal zone [4], [6], [7]. The treatment process has been improved through the development of phased arrays for FUS therapies, allowing for the treatment region to be spanned by electronic steering and eliminating the need for time consuming mechanical translation [8]–[12]. Using phased arrays with electronic steering for beam targeting provides many benefits beyond just reduced treatment time. Aberrations in the FUS beam can be corrected by calculating phase delays for each array element [13]. Phased arrays can also be used to create more complex focal shapes [14]–[16] or multi-foci patterns [10], [17]–[19] to speed up and optimize treatment. For thermal treatments, spiral sonication patterns have been proposed to ensure uniform heating of the treatment region [20], [21]. The drawback to using high-power phased arrays is that they are expensive due to the engineering challenges associated with creating phased arrays for FUS therapy [22].

Monitoring of the FUS beam’s location with respect to anatomical features is a crucial component of any FUS therapy system. MR imaging is the primary technique used for monitoring high intensity FUS therapies [23], [24]. There are many well-established clinical FUS therapy systems that employ MR imaging for therapy planning and monitoring such as those developed by InSightec [25]–[27] and Philips [28]. MR imaging can be used for localization of the FUS beam and quantitative monitoring of tissue temperatures. Although MR imaging is an effective guidance tool, it is also expensive and non-portable. Ultrasound-based techniques such as B-mode imaging [29], [30] and passive cavitation imaging (PCI) [31]–[34] have been explored as lower cost therapy monitoring tools. Our group recently developed a technique for real-time localization of an FUS beam using ultrasonic backscatter [35].

Whereas high intensity FUS pulses are used in many FUS therapies such as thermal-based treatments and histotripsy, a growing number of therapies use low intensity FUS instead. These mechanical-based therapies use low intensity FUS, often in combination with the injection of microbubbles, to induce mechanical bioeffects in the target tissue. In one work, microbubbles activated by ultrasound were used to sensitize tumors to either subsequent radiation or hyperthermia [36]–[38]. Microbubbles in tumors were activated using a 500 kHz transducer with pressure levels up to 740 kPa and a 5 minute ultrasound exposure. The microbubbles were hypothesized to activate the ceramide pathway inducing amplified cell death when afterwards exposed to radiation or hyperthermia. In a similar approach, oxygen-filled microbubbles were destroyed in tumors using a flash sequence and a diagnostic array (MI = 1.39) to improve oxygenation of hypoxic tumors resulting in improved sensitivity to radiation treatment [39]. Several groups have used low intensity ultrasound in combination with microbubbles to transiently and safely open the blood-brain barrier [40]–[44]. In these techniques MRI was used to monitor the beam location of the focused ultrasound even though low acoustic pressures and powers were utilized. Other techniques have used ultrasound with microbubbles to deliver drugs to targeted tissues, i.e., targeted drug delivery [45]. Two review articles described the use of ultrasound for neuromodulation [46], [47]. The review indicated that many different ultrasound exposure regimes provided a range of responses with many of these exposures in the diagnostic ranges of ultrasound. Hence, low intensity ultrasound has a history of successfully and safely producing neuromodulation effects. A final example of the use of low intensity FUS therapies involve the use of pulsed ultrasound to stimulate bone repair [48]. Intensity values below 100 mW/cm^2^ were used to induce improved bone healing.

Some of these low intensity FUS therapies were carried out with diagnostic imaging arrays and it is likely that many of these other therapies could be carried out using a diagnostic imaging array instead of a high power FUS source. As another example, FUS-mediated opening of the blood-brain barrier with simultaneous cavitation monitoring has been achieved using a single diagnostic imaging array [49]. Imaging arrays are attractive options for an FUS source because they allow for easy beam steering and image guidance during therapy, while also simplifying the FUS therapy system if treatment and monitoring can be carried out with a single ultrasound probe.

We describe a system for combined low intensity FUS treatment, therapy planning, and real-time qualitative monitoring using a diagnostic imaging array. This system could facilitate faster treatments, does not require the expensive high powered phased arrays used in conventional FUS therapy systems, and uses a type of ultrasound probe that is already commonplace in research and clinical settings. A mock FUS therapy was planned by acquiring a B-mode image with the imaging array and using the image to automatically design a sonication pattern. Once mock treatment was initiated, the FUS therapy beam was electronically and automatically steered throughout the treatment region. The position and size of the FUS beam was monitored in real-time using ultrasonic backscatter received by the imaging array. The FUS targeting and monitoring system was validated in a tissue-mimicking phantom and in a rat tumor *in vivo*. To verify that a diagnostic imaging array can be used as an FUS source for low intensity therapies that use microbubbles, FUS beam visualizations were reconstructed using ultrasonic backscatter from a population of microbubbles. This work is an extension of a conference paper [50]. The novelty of this work is that the efficacy of the therapy monitoring technique was evaluated using Field II simulations, further discussion on the effect of microbubbles on the therapy monitoring technique is presented, and a video of *in vivo* therapy planning was recorded.

## II. Methods

### A. Therapy Planning

The system was developed on a Verasonics Vantage 128 Ultrasound System (Kirkland, WA, USA) using an Ultrasonix L9–4/38 linear array probe (Center frequency: 5 MHz, No. elements: 128; Richmond BC, Canada). The choice of operating frequency of the transducer is important for several factors. A lower frequency allows deeper penetration of ultrasound and, therefore, deeper therapies. Higher frequency transducers allow finer control over the beam in terms of spatial resolution but with lower penetration depth. Therefore, depending on the depth of the therapy, the choice of transducer frequency is important to consider. In our study, we were assessing the capabilities of our therapy system on rat mammary tumors which were small but not very deep. Therefore, a 5-MHz transducer was used because it provided high spatial resolution and the penetration depth was not large. Furthermore, we chose the pressure output values based on experiments using the same transducer where we were able to induce detectable cavitation of microbubbles.

**Fig. 1.**
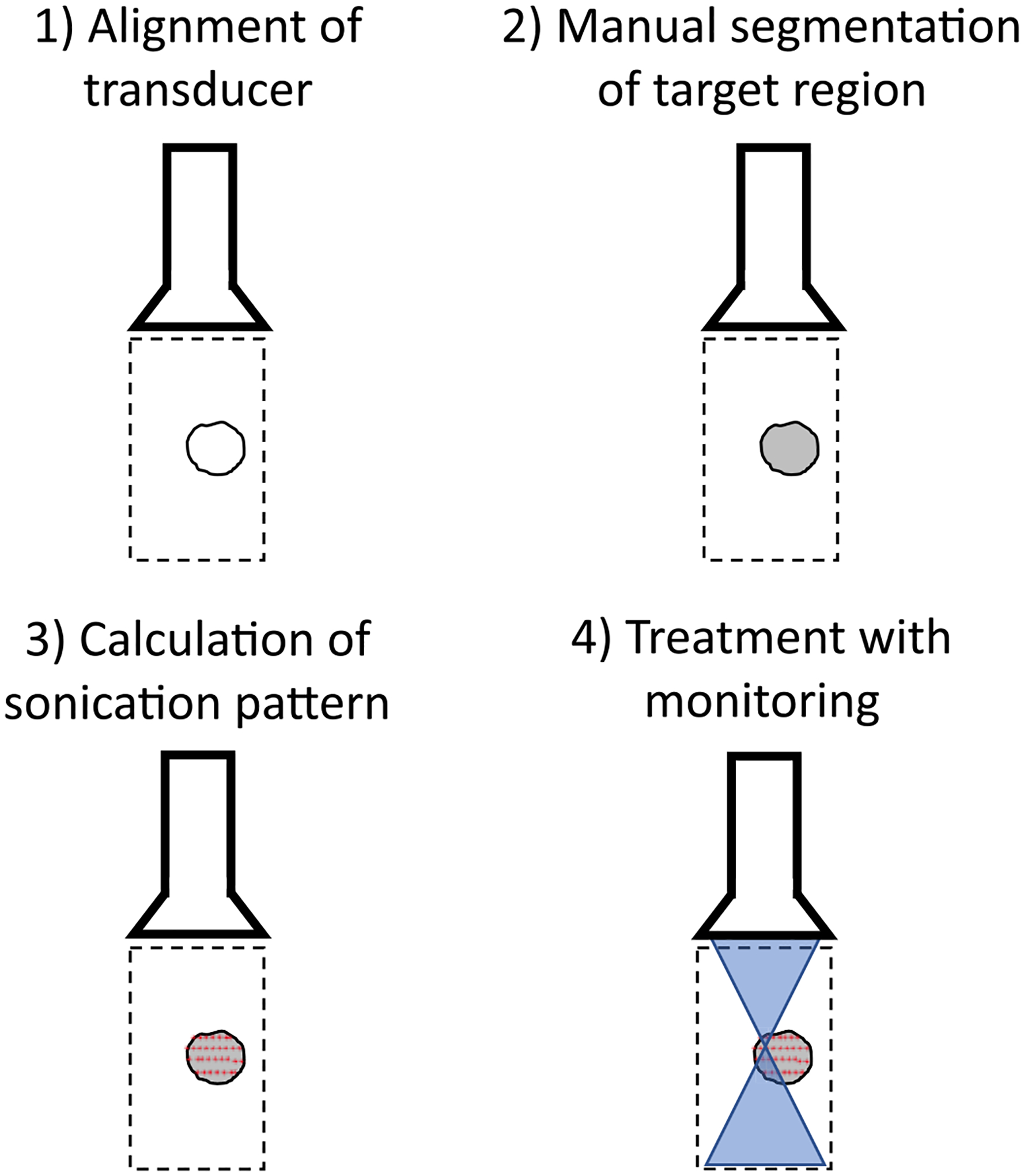
Overview of FUS therapy planning and treatment monitoring procedure using a single diagnostic imaging array. In step 1, the transducer is aligned so that an image of the target region is in the field of view. In step 2, the therapy region is manually outlined by the operator. In step 3, the target points are automatically calculated. In step 4, the beam is steered sequentially across the target region, i.e., from left to right across the points defined in the top row and then moving to the next row of points and steering the beam to each point from left to right until all rows are treated.

An overview of the therapy planning technique is shown in Figure 1. Therapy planning began by aligning the imaging array to the treatment region using real-time B-mode imaging created in the MATLAB (MathWorks; Natick, MA, USA) environment for the Verasonics system. Once alignment was complete, a B-mode image captured by the array was presented to the user for manual segmentation of the treatment region. The user then traced out the treatment region using a series of mouse clicks on the B-mode image, creating an arbitrarily shaped, closed region of interest. The system used this region of interest to calculate a bounding box, which was defined as the smallest rectangle that completely enclosed the treatment region. A grid of focal points was then generated to fill the bounding box. The lateral step size of the grid was determined using the estimated beamwidth of the FUS beam and the axial step size was determined using the estimated depth of field. The estimated −3 dB transmit beamwidth *R*_−3*d B*_ and depth of field *DOF*_−3*d B*_ of a rectangular focusing source with a continuous wave excitation can be approximated as:

(1) $$R_{-3dB}=0.886\cdot \lambda \cdot f_{\text{\#}}$$

and

(2) $$DOF_{-3dB}=7.1\cdot \lambda \cdot {\left(f_{\text{\#}}\right)}^{2},$$

where *λ* is the wavelength of the excitation and *f*_#_ is the f-number of the FUS beam [51]. The *f*_#_ of the FUS beam was determined for each treatment region by taking the ratio of the depth of the region’s center point to the lateral diameter of the imaging array. The lateral step size Δ*x* and axial step size Δ*z* of the focal point grid were empirically defined as:

(3) $$\Delta x=R_{-3dB}$$

and

(4) $$\Delta z=0.5\cdot DOF_{-3dB},$$

where the *x*-axis is the lateral direction along the array and the *z*-axis is the axial direction perpendicular to the array. Every other row of focal points was offset from the left edge of the bounding box by 0.5 · Δ*x* in the positive *x* direction. This reduced the overlap between the focal zones of the FUS beams in adjacent rows. The focal points in the bounding box that did not fall within the region of interest were discarded, which resulted in a grid of focal points that evenly filled the treatment region. Once the selected treatment region was confirmed by the user, the Versasonics script resumed and FUS treatment with monitoring was immediately started using a focused beam targeted to the first point in the grid. The Verasonics script then automatically updated the focal point to move through the focal point grid for treatment of the entire region.

### B. Beam Monitoring

**Fig. 2.**
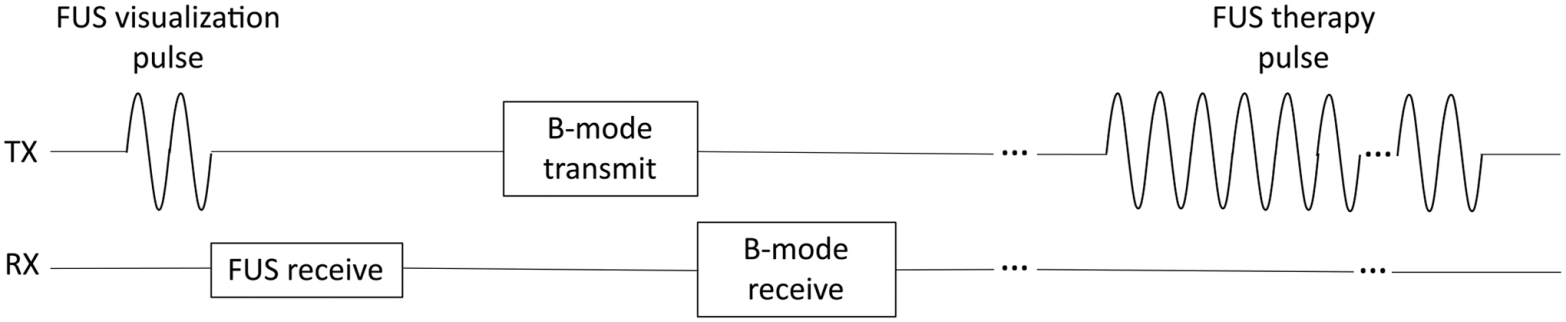
Excitation sequence for combined FUS beam monitoring and FUS treatment using a single diagnostic imaging array.

An overview of the excitation sequence executed by the imaging array for low intensity FUS treatment with simultaneous monitoring is shown in Figure 2. This excitation sequence occurs in step 4 of the treatment planning sequence outlined in Figure 1. Beam monitoring was conducted during the off-cycle of the FUS treatment by reconstructing the intensity field of the FUS beam using backscatter from a focused visualization pulse [35]. During the beam monitoring phase, the imaging array transmitted a focused visualization pulse to match the focal properties of the therapy excitation and the real backscattered RF data from this pulse were recorded by the array at approximately four samples per wavelength. All beam visualization utilized the fundamental band of the transducer and did not use harmonics for beam reconstruction. A delay- and-sum (DAS) with generalized coherence factor (GCF) weighting [52] beamformer was used to process the data. Dynamic receive focusing was used during beamforming and a dynamic width receive aperture (*f*_#_ = 1) was used to maintain constant *f*_#_ imaging. The beamformer calculated time delays *τ*[*x*, *z*] for each point [*x*, *z*] using:

(5) $$\tau [x,z]=\tau_{F}\left[x_{c}\right]+\frac{z+\sqrt{z^{2}+{\left(x-x_{c}\right)}^{2}}}{c}$$

where *c* is the speed of sound in the medium and *τ*_*F*_[*x*_*c*_] is the time delay applied on transmit to focus the center element *x*_*c*_ in the receive subaperture.

GCF weighting was used to improve lateral resolution and reduce sidelobes in the FUS beam reconstructions. The GCF is a measure of signal integrity and is a generalization of the original coherence factor (CF) [53], [54]. The GCF is expressed as:

(6) $$GCF[x,z]=\frac{\sum_{k=-M}^{M}{\left|S_{\left[x,z\right]}[k]\right|}^{2}}{N\sum_{k=0}^{N-1}{\left|s_{\left[x,z\right]}[k]\right|}^{2}},$$

where *s*_[*x*,*z*]_(*k*) is the receive subaperture RF data after applying time delays for the point [*x*, *z*], *S*_[*x*,*z*]_[*k*] is the discrete Fourier transform of *s*_[*x*,*z*]_[*k*], *N* is the number of channels in the receive subaperture, and *M* is a parameter of the GCF (*M* = 2 was used). The GCF can be interpreted as a ratio of the energy contained in low frequency and DC components of the subaperture data to the total energy of the subaperture data.

The output *y*[*x*, *z*] of the DAS-GCF beamformer was:

(7) $$y[x,z]=GCF[x,z]\overset{N-1}{\underset{i=0}{\sum }}w[i]s_{\left[x,z\right]}[i]$$

where and *w*[*i*] is a Hanning window of length *N*.

The scattering properties of the medium affected the FUS beam reconstruction because it was produced using ultrasonic backscatter. For example, the FUS beam reconstruction would appear brighter in highly scattering areas, even if the incident intensity field of the FUS beam remained constant. To address this, a spatially registered B-mode image was captured with the imaging array after transmitting the focused visualization pulse and was used to normalize the FUS beam reconstruction. This process was referred to as normalizing by echogenicity, and it was an attempt to equalize the FUS beam reconstruction across regions having different echogenicity. The normalization factor *λ*[*x*, *z*] was calculated as follows:

(8) $$\lambda [x,z]=\frac{B_{max}}{B[x,z]}$$

where *B*[*x*, *z*] is the smoothed envelope detected B-mode image (filtered with a 5.5 wavelengths × 7 wavelengths moving average kernel) and *B*_*max*_ is the maximum value of *B*[*x*, *z*]. The B-mode image was smoothed to ensure that small features and speckle noise did not affect the normalization procedure. The size of the averaging kernel was empirically determined and was selected to minimize the mean square error between normalized intensity field reconstructions captured in heterogeneous regions and baseline intensity field reconstructions captured in homogeneous regions [35].

The normalization factor was applied to the beamformed data *y*[*x*, *z*] to produce *y*_*norm*_[*x*, *z*]. The intensity field *I*[*x*, *z*] of the FUS beam was then estimated using a pulse intensity integral, which can be used to find the intensity of an acoustic waveform given the pressure. A sliding integral was approximated at each point in the beamformed data *y*_*norm*_[*x*, *z*] to estimate the intensity of the backscattered signal from each point:

(9) $$I[x,z]=\overset{L-1}{\underset{i=0}{\sum }}\Delta z{\left|y_{\text{norm}}[x,z+i]\right|}^{2}$$

where *L* is the length of the transmit pulse in samples and Δ*z* is the axial sampling period. The normalization factor was not applied to points where the decibel scale value of *B*[*x*, *z*] was 60 dB or more below *B*_*max*_ because it was assumed that the signal from these areas was comprised of mostly noise. After applying the normalization factor, the FUS intensity field reconstruction *I*[*x*, *z*] was converted to decibel scale, normalized to the maximum, and overlaid onto the co-aligned B-mode image for display.

### C. Excitation Sequence

The same linear array used for therapy planning was used for the therapy excitation and beam monitoring. The imaging array was driven by a Verasonics Vantage 128 Research Ultrasound System. Data were collected from a tissue-mimicking phantom (Supertech Model ATS 539; Elkhart, IN, USA) and a rat tumor *in vivo*.

The excitation sequence was split into two phases: therapy planning and treatment with real-time monitoring. The treatment portion of the excitation sequence was based on a low intensity radiotherapy sensitization FUS therapy [36]. However, no therapeutic effects were induced *in vivo* because this FUS therapy requires the use of microbubbles, which were not used in the phantom or *in vivo* portions of this study. Hence, the excitation sequence is referred to as a mock FUS therapy. During the therapy planning phase, the imaging array used 11 steered plane waves (−18° to 18°) for real-time B-mode imaging using coherent plane wave compounding [55]. Once the imaging array was aligned to the treatment region, a B-mode image was manually segmented by the user and a focal point grid was created. During the treatment with monitoring phase, the imaging array carried out B-mode imaging and FUS beam visualization during the off-cycle of the mock FUS therapy. Each treatment window was 50 ms long and was followed by a 2 s imaging and beam visualization window. The imaging window was relatively long in duration because during an actual FUS therapy it would provide time for microbubbles to refresh in the focal region. The FUS therapy pulse was a 160-cycle tone burst at 5 MHz, operated at a pulse repetition frequency of 3 kHz for each 50 ms treatment window. The therapy beam was re-targeted to the next point in the focal point grid following each treatment and imaging window. For the 2 s imaging window, the imaging array first transmitted a 2-cycle, 5 MHz focused visualization pulse targeted to the same location as the FUS therapy beam. The imaging array then acquired a B-mode image with the coherent plane wave compounding sequence used during therapy planning. The FUS beam visualization and B-mode imaging sequences were repeated for the duration of the 2 s imaging window. The focused visualization pulse had a mechanical index (MI) of 0.54 and the FUS therapy pulse had an MI of 0.78. The MI values were measured using a membrane hydrophone (Element diameter: 0.04 mm; Precision Acoustics, Dorchester UK) in degassed water. After a treatment and imaging window at one focal point, the imaging array was re-focused to the next focal point in the grid. This sequence of interleaved treatment and imaging was repeated for all points in the focal point grid.

To examine possible beam variations between the actual therapy excitation using 160 cycles and the visualization excitation of 2 cycles, Field II was used to produce beam plots of the two excitation configurations [56], [57]. The beamwidth and depth of field for the two excitations were then compared.

### D. Animal Experiment

All animal experiments were approved by the Institutional Animal Care and Use Committee at the University of Illinois at Urbana-Champaign. Tumors were induced in the mammary fat pad of a female F344 rats by injecting MAT tumor cells (5 × 10^2^ cells in 100 *μ*L) on each side of the abdomen. Once the tumors grew to 5 – 15 mm in diameter, the animals were anesthetized using isoflurane and imaged. During imaging, a bucket of degassed water with a bottom made of thin plastic film was placed over the animal’s body after mineral oil was applied to the tumors for acoustic coupling. The degassed water allowed for a 1 – 2 cm standoff distance between the imaging array and the tumors.

### E. Microbubble Experiment

The beam visualization technique was evaluated in the presence of microbubbles to determine if it would be suitable for use in microbubble-based FUS therapies. A latex balloon was filled with 3 mL of Definity microbubbles diluted in 40 mL of distilled water. The Definity microbubbles were activated using a Vialmix activation device one week prior to the experiment and were re-agitated by hand immediately before use. The physical properties of Definity microbubbles and similar lipid-shelled microbubbles remain stable for up to two weeks [58], [59]. The balloon was then submerged in degassed water and the beam visualization excitation sequence was carried out with an FUS beam focused to a point in the balloon.

## III. Results

**Fig. 3.**
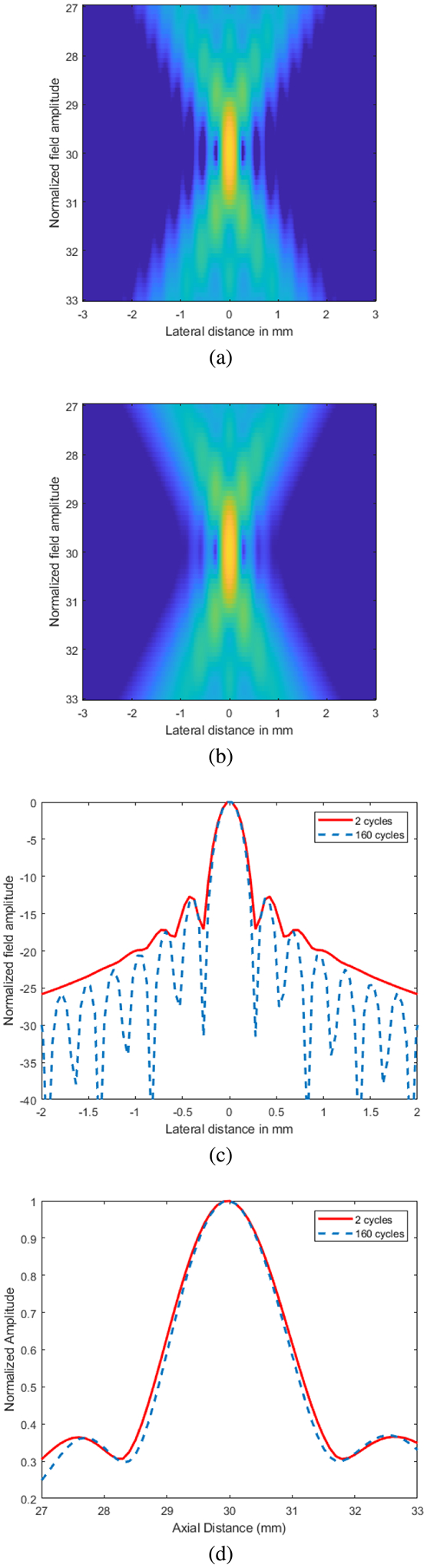
Beam plots for (a) 160-cycle excitation and the (b) 2-cycle excitation along with cross sections representing the (c) the beamwidth at the focus and (d) the depth of field for the two excitations.

The results of the Field II simulation of the focused therapy beam produced using 160 cycles and the focused visualization beam using 2 cycles is shown in Figure 3. The beam plots for the 160-cycle excitation and the 2-cycle excitation are similar in appearance. From the beam plot using the 2-cycle excitation, a smearing of the sidelobes can be observed. The beamwidth taken laterally across the focus indicates that the beam generated with the 2-cycle excitation results in a negligible increase in the beam width and smoothing of the sidelobes. The dept of field also resulted in minimal increase in the 2-cycle excitation versus the 160-cycle excitation. Therefore, based on the simulation results, the visualization based on the 2-cycle excitation was a good representation of the therapy beam shape and size.

**Fig. 4.**
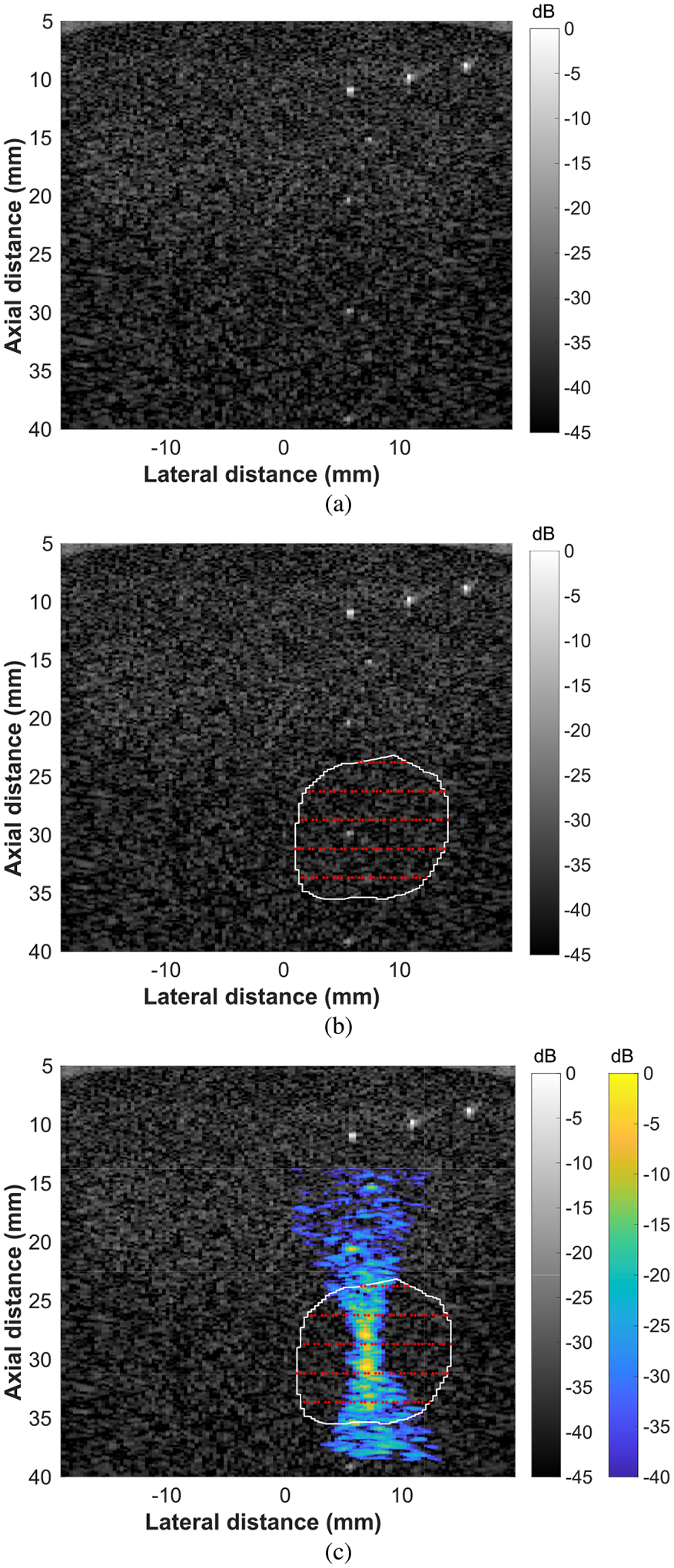
Demonstration of therapy planning procedure in a tissue-mimicking phantom. (a) B-mode image used for alignment. (b) Manually segmented B-mode image with user-input treatment region. The treatment region is outlined with a white line and the focal point grid is depicted with red points. (c) B-mode image with FUS beam visualization overlay (blue/yellow colormap). The ultrasound beam was focused at a focal point near the center of the region of interest, and the point was in the third row from the top of the focal point grid.

The therapy planning and treatment monitoring system was tested in a tissue-mimicking phantom (Figure 4). First, B-mode imaging was used to align the imaging array to a series of a point targets (Figure 4a). Next, the B-mode image was manually segmented by the user and a grid of focal points was automatically calculated to fill the treatment region (Figure 4b). The focal points were not all equally spaced in the lateral direction because a discrete grid was used for the calculations, which resulted in small rounding errors. Finally, a mock FUS treatment was initiated and the FUS beam was monitored in real-time using ultrasonic backscatter (Figure 4c).

**Fig. 5.**
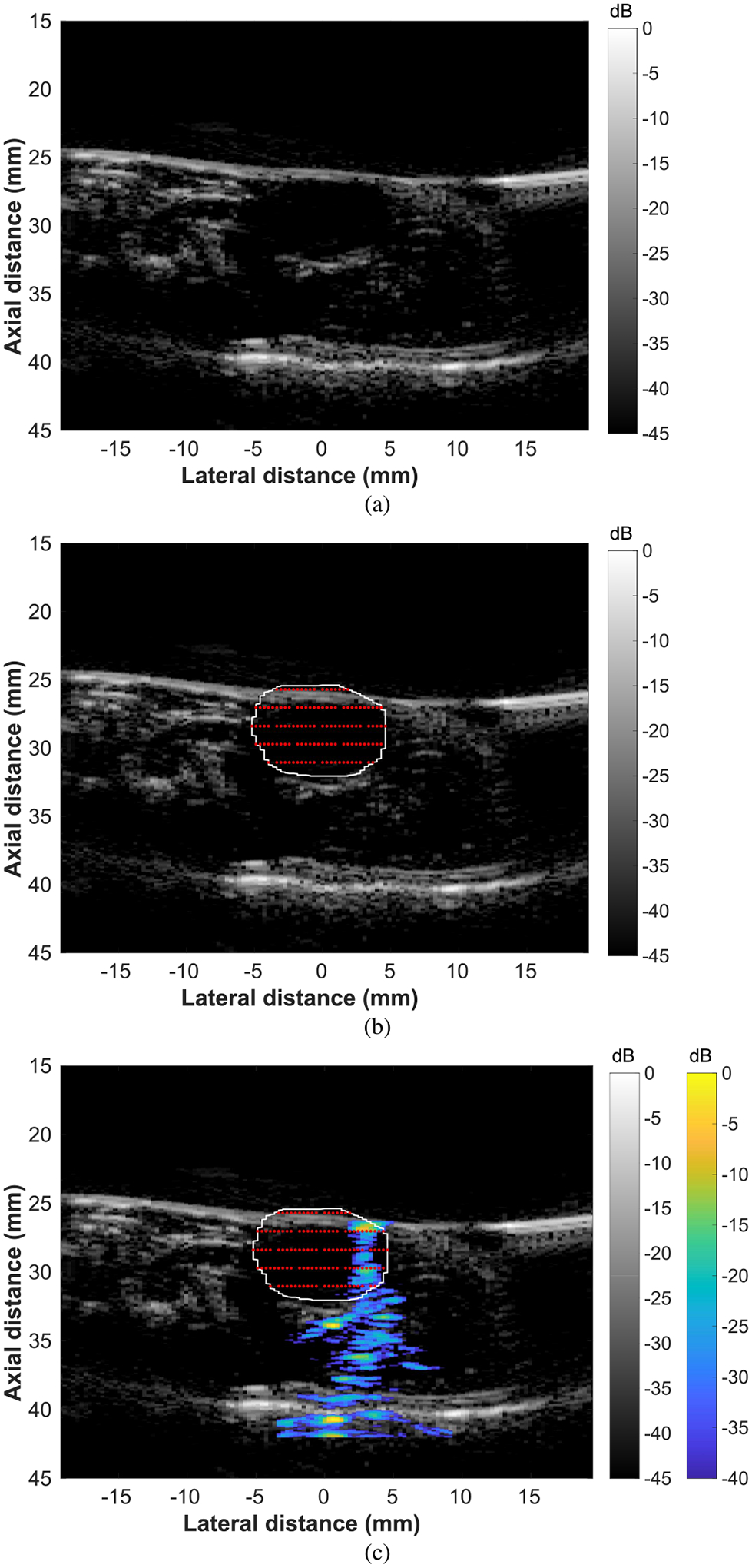
Demonstration of therapy planning procedure in a rat tumor *in vivo*. (a) B-mode image used for alignment. (b) Manually segmented B-mode image with user-input treatment region. The treatment region is outlined with a white line and the focal point grid is depicted with red points. (c) B-mode image with FUS beam visualization overlay (blue/yellow colormap). The ultrasound beam was focused at a focal point near the right edge of the tumor, and the point was in the third row from the top of the focal point grid.

The therapy planning and treatment monitoring system was also tested *in vivo*. An overview of the planning and monitoring of a mock FUS treatment in a rat tumor *in vivo* is shown in Figure 5. The tumor was manually segmented from a B-mode image and a grid of focal points was automatically calculated. The excitation sequence for a radiosensitization FUS treatment was then administered while the position of the FUS beam was monitored in real-time. The FUS beam can clearly be localized near the right edge of the tumor and targeted to a focal point in the third row of the focal point grid. The FUS beam was not visualized at depths from 10–25 mm because this area was the degassed water standoff, which did not scatter enough of the focused visualization pulse to reconstruct the FUS beam. Furthermore, there were regions below the tumor, i.e., the outlined region, where backscattered signal was in the noise and was therefore not amplified in the normalization factor. This resulted in drop out of the beam visualization below the tumor.

**Fig. 6.**
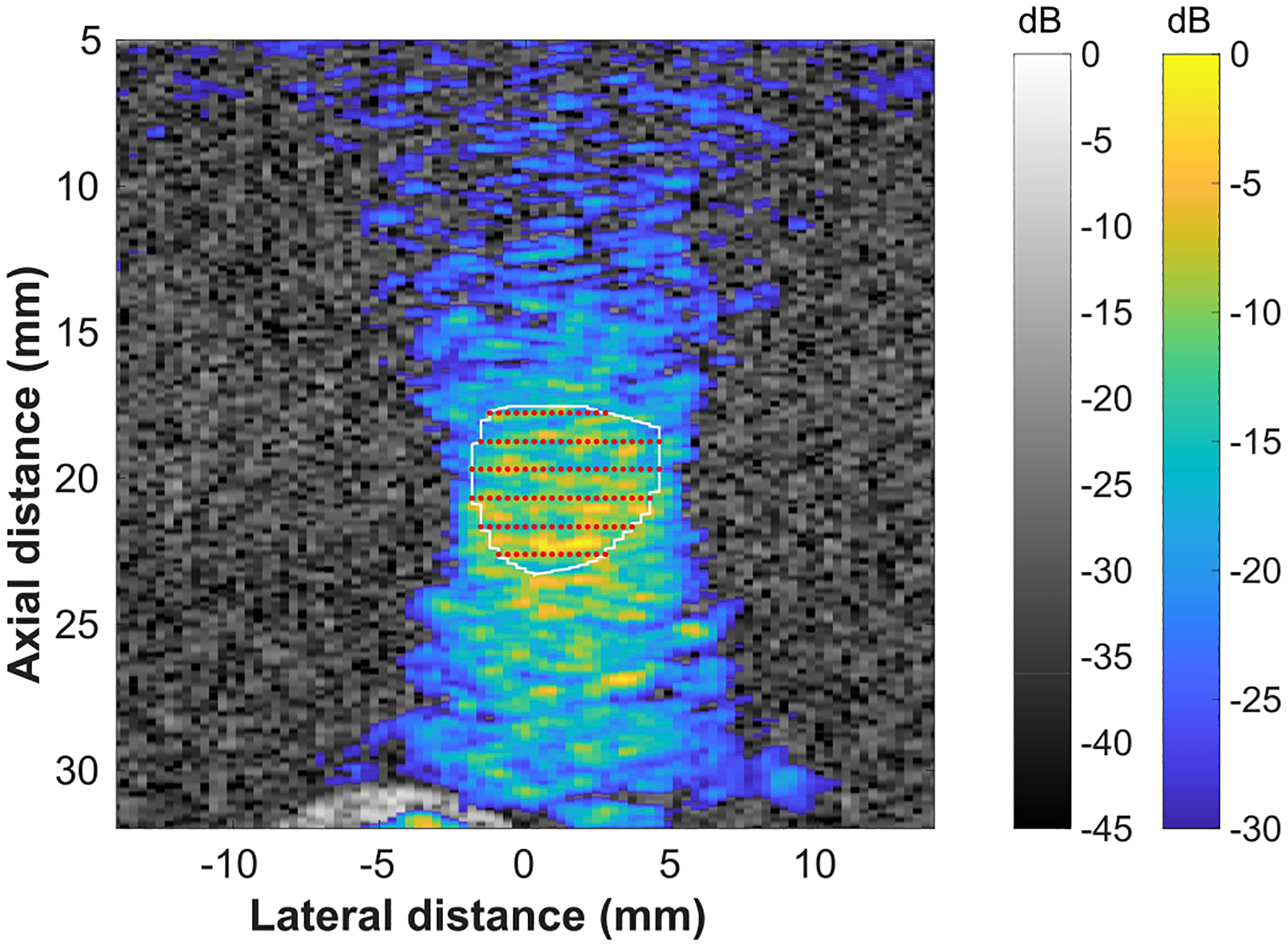
Average intensity field delivered to a treatment region during a mock FUS therapy in a tissue-mimicking phantom. The treatment region is outlined with a white line and the focal point grid is depicted with red points.

To visualize the intensity field delivered to a treatment region over an entire FUS therapy, a mock FUS therapy was carried out in a tissue-mimicking phantom and the intensity fields captured at every point in the focal point grid were averaged (Figure 6). The average intensity field completely filled the treatment region. A minimal amount of energy was visualized outside of the treatment region.

**Fig. 7.**
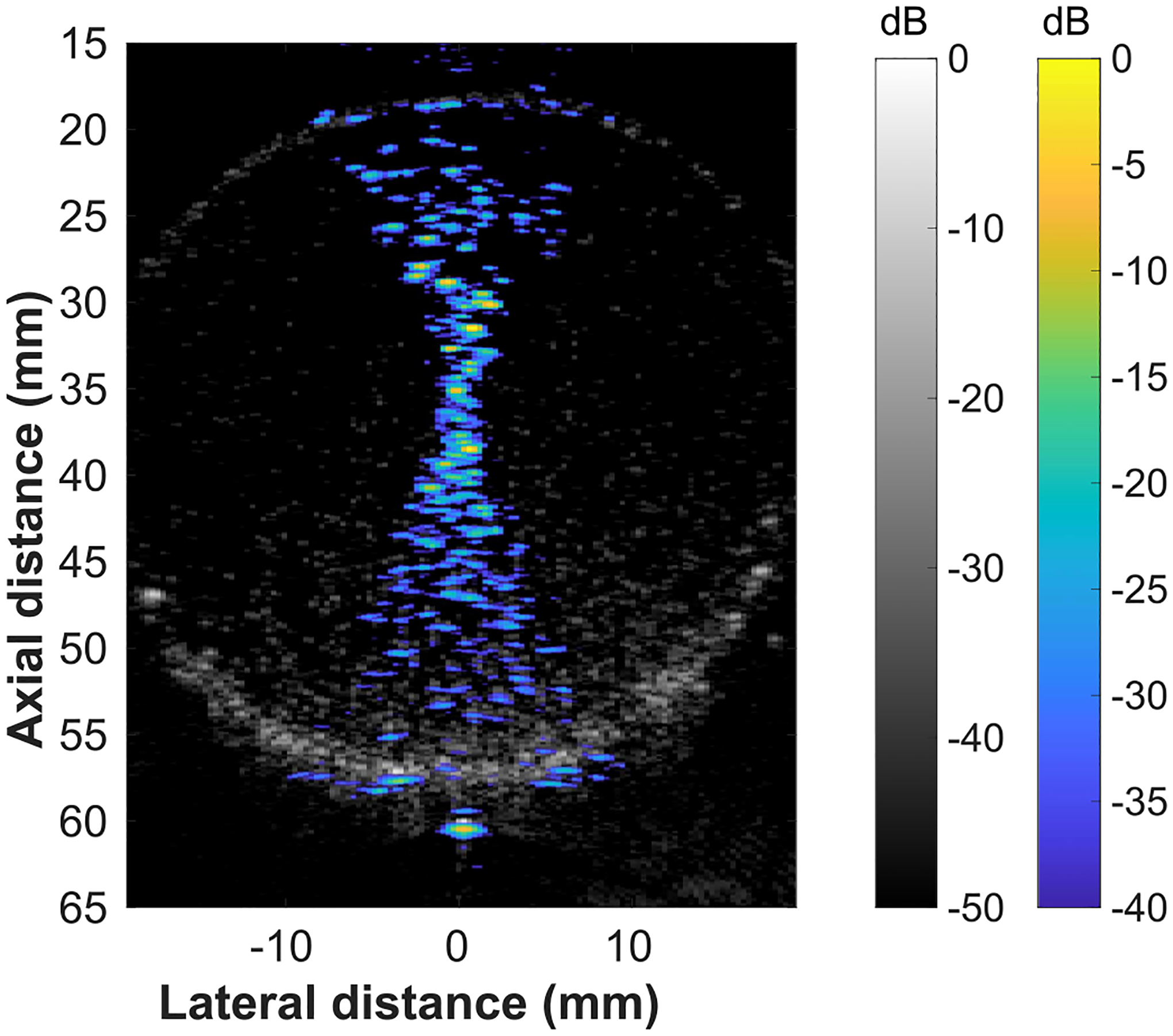
FUS beam visualization captured in a balloon containing definity microbubbles in distilled water. The FUS beam was focused to 36 mm axially and 0 mm laterally.

To determine if the microbubble interactions would degrade the therapy monitoring in a microbbule-based FUS treatment, an FUS beam visualization was captured in a balloon containing Definity microbubbles (Figure 7). The FUS beam can be clearly visualized within the balloon using backscatter from the microbubbles. The microbubbles were concentrated near the bottom of the balloon due to radiation force from the FUS beam.

A video depicting therapy planning and real-time FUS beam visualization in a rat tumor *in vivo* can be found in the supplementary media files. At the beginning of the video, a mock FUS treatment is being administered to a rat tumor. The intensity field reconstruction of the FUS beam is overlaid onto a co-aligned B-mode image and the treatment region is outlined with a white line. The mock treatment is then paused, and a new treatment region is segmented from a B-mode image. The ability to pause treatment and design a new therapy plan could be used to correct for tissue motion or movement of the transducer during a real FUS treatment. The mock FUS treatment is then resumed with the updated treatment plan and the imaging array is re-targeted to the first point in the sonication pattern. The FUS beam is then monitored as it is scanned through the treatment region.

## IV. Discussion

A diagnostic imaging array and an ultrasound research platform (Verasonics Vantage) were used for combined therapy planning, real-time monitoring, and low intensity FUS treatment. The GCF was used to reduce the effects of sidelobes on the reconstruction and registered B-mode images were used to partially account for variations in the intensity of the beam reconstruction. The use of the GCF provided a reduction in the sidelobes observed in the beam visualization. However, the use of different sorts of windows, other than the Hanning window, on the transmitted focused beam could provide improved reduction of sidelobes in the beam visualization. Additional windowing options could be explored in future work. The therapy planning and FUS beam monitoring were tested in a tissue mimicking phantom (Figure 4) and in a rat tumor *in vivo* (Figure 5). The FUS tone burst had an MI of 0.78, which has been demonstrated to be more than sufficient to activate microbubbles for therapy *in vivo* [60]. The intensity field delivered to a treatment region over a therapy session was visualized to confirm that the therapy planning technique resulted in complete treatment of the target region (Figure 6). Finally, an FUS beam visualization was captured in a balloon containing microbubbles (Figure 7). This result suggests that the proposed therapy monitoring technique could be used in microbubble-based treatments.

Implementing therapy planning on the same transducer that is used for FUS treatment facilitates fast and simplified FUS therapies where no registration between different ultrasound probes or imaging modalities is required. The focal point grid created during therapy planning was designed to evenly fill the entire treatment region, but it is not necessarily optimal for low intensity FUS treatments. Further study on the effect of the sonication pattern on treatment efficacy would need to be conducted to determine what type of focal point pattern would be ideal. For example, spiral sonication patterns have been proposed as the ideal focal point pattern for thermal-based FUS therapies [20], [21].

The FUS beam visualization technique can provide real-time qualitative monitoring of an FUS beam’s position and size, but in some applications quantitative monitoring of cavitation emissions or tissue temperature is also required. In these cases, FUS beam visualization using ultrasonic backscatter could supplement quantitative monitoring and provide additional useful information. When selecting an imaging array to use for a combined monitoring, planning, and treatment system, there is a trade-off between selecting a high frequency array to maximize the image resolution for therapy monitoring and selecting a low frequency array for better FUS treatment. A high frequency FUS source must transmit higher pressure values to reach a given MI and will be more affected by frequency-dependent attenuation as compared to a low frequency FUS source. The FUS beam visualization technique used here could also be used in a more conventional high intensity FUS system with a separate imaging array and FUS source. However, the monitoring technique assumes that the low MI, short pulse length FUS visualization beam is representative of the FUS therapy beam. For high intensity FUS treatments, this assumption may break down due to non-linear propagation of the FUS therapy beam. Ideally, a 2-dimensional (2D) imaging array should be used for this system because it could be used to plan a 3D therapy by manually segmenting multiple image slices along the elevational plane. To treat a 3D volume using a linear array such as the one used in this study, one would need to mechanically scan the array along the elevational plane.

The B-mode was constructed using a coherent plane wave compounding beamformer and an imaging pulse with constant f-number focusing on receive. This beamformer was used for B-mode imaging because constant f-number plane wave compounding minimizes the spatial changes caused by the beam diffraction pattern as compared to the DAS-GCF beamformer used for beam visualization. Hence, the B-mode image is considered a map of intensity of scattering from the field. However, the use of a different focusing parameter on transmit for the beam visualization pulse would result in a different resolution cell and differences in the spatial variation of intensity or speckle. Therefore, the use of the B-mode for correcting for intensity variations is not a perfect correction, as can be observed in the *in vivo* data. Because we are only using the fundamental band in both B-mode and beam visualization, the nonlinear effects introduced by objects like microbubbles would mainly be observed in changes in brightness due to bubble scattering, which would be true for both the B-mode and visualization pulses. The nonlinear scattering from the bubble means that the corrective term from the B-mode will be less effective when bubbles are present and when the B-mode imaging pulse and beam visualization pulse have widely varying pressures. In the end, the correction is meant to partially account for differences in scattering and structure that would suppress the beam visualization and allow the beam to be visualized in image regions with low intensity regions compared to brighter regions. Additional approaches could be implemented to improve the correction and have improved visualization when in settings like *in vivo*.

There are a large number of low intensity FUS therapies, and the exact physical mechanisms that produce therapeutic effects are not well-characterized for many treatments. Thus, this system may not be suitable for all low intensity FUS treatments. However, the MIs achievable with a diagnostic array like the one used in this work suggest that an imaging array should be capable of administering low intensity FUS treatments that use stable cavitation such as transient opening of the blood-brain barrier, for which stable cavitation is the physical mechanism at low to moderate pressures [61].

## V. Conclusion

In this study, a system for therapy planning, real-time therapy monitoring, and low intensity FUS treatment using a single diagnostic imaging array was presented. Therapy planning was carried out by manually segmenting a B-mode image and FUS beam monitoring was done by reconstructing the intensity field of the FUS beam using ultrasonic backscatter as it scanned through the segmented treatment region. Therapy planning and FUS beam visualization were demonstrated in a tissue-mimicking phantom and in a rat tumor *in vivo*. Implementing all components of an FUS therapy using a single transducer could enable faster and more simplified low intensity FUS treatments in both research and clinical settings. In future work, this system could be used to administer an *in vivo* FUS therapy and the therapy outcomes could be compared with the outcomes for conventional FUS systems.

## Supplementary Material

supp1-3140176

## References

[1] Ebbini ES and Haar GRT, “Ultrasound-guided therapeutic focused ultrasound: Current status and future directions,” Int. J. Hyperthermia, vol. 31, no. 2, pp. 77–89, Mar. 2015. 25614047 10.3109/02656736.2014.995238

[2] Izadifar Z, Izadifar Z, Chapman D, and Babyn P, “An introduction to high intensity focused ultrasound: Systematic review on principles, devices, and clinical applications,” J. Clin. Med, vol. 9, no. 2, p. 460, Feb. 2020. 10.3390/jcm9020460 PMC7073974 32046072

[3] Zhou M, Chen J-Y, Tang L-D, Chen W-Z, and Wang Z-B, “Ultrasound-guided high-intensity focused ultrasound ablation for adenomyosis: The clinical experience of a single center,” Fertility Sterility, vol. 95, no. 3, pp. 900–905, Mar. 2011. 21067723 10.1016/j.fertnstert.2010.10.020

[4] Hynynen K , “MR imaging-guided focused ultrasound surgery of fibroadenomas in the breast: A feasibility study,” Radiology, vol. 219, pp. 176–185, Apr. 2001, doi: 10.1148/radiology.219.1.r01ap02176. 11274554

[5] Clement G and Hynynen K, “A non-invasive method for focusing ultrasound through the human skull,” Phys. Med. Biol, vol. 47, no. 8, pp. 1219–1236, Apr. 2002, doi: 10.1088/0031-9155/47/8/301. 12030552

[6] Sanghvi NT , “Noninvasive surgery of prostate tissue by high intensity focused ultrasound: An updated report,” Eur. J. Ultrasound, vol. 9, no. 1, pp. 19–29, Mar. 1999. 10099163 10.1016/s0929-8266(99)00010-5

[7] Wu F , “Pathological changes in human malignant carcinoma treated with high-intensity focused ultrasound,” Ultrasound Med. Biol, vol. 27, no. 8, pp. 1099–1106, Aug. 2001. [Online]. Available: http://www.sciencedirect.com/science/article/pii/S0301562901003891 11527596 10.1016/s0301-5629(01)00389-1

[8] Goss SA, Frizzell LA, Kouzmanoff JT, Barich JM, and Yang JM, “Sparse random ultrasound phased array for focal surgery,” IEEE Trans. Ultrason., Ferroelectr., Freq. Control, vol. 43, no. 6, pp. 1111–1121, Nov. 1996.

[9] Fjield T and Hynynen K, “The combined concentric-ring and sector-vortex phased array for MRI guided ultrasound surgery,” IEEE Trans. Ultrason., Ferroelectr., Freq. Control, vol. 44, no. 5, pp. 1157–1167, Sep. 1997.

[10] Hand JW, Shaw A, Sadhoo N, Rajagopal S, Dickinson RJ, and Gavrilov LR, “A random phased array device for delivery of high intensity focused ultrasound,” Phys. Med. Biol, vol. 54, no. 19, pp. 5675–5693, Oct. 2009, doi: 10.1088/0031-9155/54/19/002. 19724099

[11] Lu M, Wan M, Xu F, Wang X, and Chang X, “Design and experiment of 256-element ultrasound phased array for noninvasive focused ultrasound surgery,” Ultrasonics, vol. 44, pp. e325–e330, Dec. 2006. 16949119 10.1016/j.ultras.2006.07.015

[12] Hynynen K , “500-element ultrasound phased array system for noninvasive focal surgery of the brain: A preliminary rabbit study with *ex vivo* human skulls,” Magn. Reson. Med, vol. 52, no. 1, pp. 100–107, Jul. 2004. 15236372 10.1002/mrm.20118

[13] Kyriakou A, Neufeld E, Werner B, Paulides MM, Szekely G, and Kuster N, “A review of numerical and experimental compensation techniques for skull-induced phase aberrations in transcranial focused ultrasound,” Int. J. Hyperthermia, vol. 30, no. 1, pp. 36–46, 2014, doi: 10.3109/02656736.2013.861519. 24325307

[14] Cain CA and Umemura S, “Concentric-ring and sector-vortex phased-array applicators for ultrasound hyperthermia,” IEEE Trans. Microw. Theory Techn, vol. MTT-34, no. 5, pp. 542–551, May 1986.

[15] Pulkkinen A, Huang Y, Song J, and Hynynen K, “Simulations and measurements of transcranial low-frequency ultrasound therapy: Skull-base heating and effective area of treatment,” Phys. Med. Biol, vol. 56, no. 15, pp. 4661–4683, Aug. 2011. 21734333 10.1088/0031-9155/56/15/003

[16] McGough RJ, Ebbini ES, and Cain CA, “Direct computation of ultrasound phased-array driving signals from a specified temperature distribution for hyperthermia,” IEEE Trans. Biomed. Eng, vol. 39, no. 8, pp. 825–835, Aug. 1992. 1505996 10.1109/10.148390

[17] Ibbini MS and Cain CA, “A field conjugation method for direct synthesis of hyperthermia phases-array heating patterns,” IEEE Trans. Ultrason., Ferroelectr., Freq. Control, vol. 36, no. 1, pp. 3–9, Jan. 1989. 18284943 10.1109/58.16962

[18] Hertzberg Y, Naor O, Volovick A, and Shoham S, “Towards multifocal ultrasonic neural stimulation: Pattern generation algorithms,” J. Neural Eng, vol. 7, no. 5, Aug. 2010, Art. no. 056002, doi: 10.1088/1741-2560/7/5/056002. 20720281

[19] Partanen A, Tillander M, Yarmolenko PS, Wood BJ, Dreher MR, and Köhler MO, “Reduction of peak acoustic pressure and shaping of heated region by use of multifoci sonications in MR-guided high-intensity focused ultrasound mediated mild hyperthermia,” Med. Phys, vol. 40, no. 1, Dec. 2012, Art. no. 013301, doi: 10.1118/1.4769116. PMC3537758 23298120

[20] Lele PP, “Induction of deep, local hyperthermia by ultrasound and electromagnetic fields: Problems and choices,” Radiat. Environ. Biophys, vol. 17, no. 3, pp. 205–217, Sep. 1980. 7443976 10.1007/BF01323647

[21] Mougenot C, Salomir R, Palussière J, Grenier N, and Moonen CT, “Automatic spatial and temporal temperature control for MR-guided focused ultrasound using fast 3D MR thermometry and multispiral trajectory of the focal point,” Magn. Reson. Med, vol. 52, no. 5, pp. 1005–1015, 2004, doi: 10.1002/mrm.20280. 15508173

[22] Hynynen K and Jones RM, “Image-guided ultrasound phased arrays are a disruptive technology for non-invasive therapy,” Phys. Med. Biol, vol. 61, no. 17, pp. R206–R248, Aug. 2016, doi: 10.1088/0031-9155/61/17/R206. 27494561 PMC5022373

[23] Hynynen K , “A clinical, noninvasive, MR imaging-monitored ultrasound surgery method,” RadioGraphics, vol. 16, no. 1, pp. 185–195, Jan. 1996. 10946699 10.1148/radiographics.16.1.185

[24] Jolesz FA, “MRI-guided focused ultrasound surgery,” Annu. Rev. Med, vol. 60, pp. 417–430, Feb. 2009. 19630579 10.1146/annurev.med.60.041707.170303 PMC4005559

[25] Elias WJ , “A pilot study of focused ultrasound thalamotomy for essential tremor,” New England J. Med, vol. 369, pp. 640–648, Aug. 2013. 23944301 10.1056/NEJMoa1300962

[26] Napoli A , “Real-time magnetic resonance–guided high-intensity focused ultrasound focal therapy for localised prostate cancer: Preliminary experience,” Eur. Urol, vol. 63, no. 2, pp. 395–398, Feb. 2013. 23159454 10.1016/j.eururo.2012.11.002

[27] Tempany CMC, Stewart EA, McDannold N, Quade BJ, Jolesz FA, and Hynynen K, “MR imaging–guided focused ultrasound surgery of uterine leiomyomas: A feasibility study,” Radiology, vol. 226, no. 3, pp. 897–905, Mar. 2003. 12616023 10.1148/radiol.2271020395

[28] Köhler MO , “Volumetric HIFU ablation under 3D guidance of rapid MRI thermometry,” Med. Phys, vol. 36, no. 8, pp. 3521–3535, Aug. 2009. 19746786 10.1118/1.3152112

[29] Vaezy S , “Real-time visualization of high-intensity focused ultrasound treatment using ultrasound imaging,” Ultrasound Med. Biol, vol. 27, no. 1, pp. 33–42, Jan. 2001. 11295268 10.1016/s0301-5629(00)00279-9

[30] Khokhlova TD , “Ultrasound-guided tissue fractionation by high intensity focused ultrasound in an *in vivo* porcine liver model,” Proc. Nat. Acad. Sci. USA, vol. 111, no. 22, pp. 8161–8166, Jun. 2014. 24843132 10.1073/pnas.1318355111 PMC4050569

[31] Salgaonkar VA, Datta S, Holland CK, and Mast TD, “Passive cavitation imaging with ultrasound arrays,” J. Acoust. Soc. Amer, vol. 126, no. 6, pp. 3071–3083, Dec. 2009. 20000921 10.1121/1.3238260 PMC2803721

[32] Gyöngy M and Coussios C-C, “Passive spatial mapping of inertial cavitation during HIFU exposure,” IEEE Trans. Biomed. Eng, vol. 57, no. 1, pp. 48–56, Jan. 2010. 19628450 10.1109/TBME.2009.2026907

[33] Coviello C , “Passive acoustic mapping utilizing optimal beamforming in ultrasound therapy monitoring,” J. Acoust. Soc. Amer, vol. 137, no. 5, pp. 2573–2585, May 2015, doi: 10.1121/1.4916694. 25994690

[34] Haworth KJ, Bader KB, Rich KT, Holland CK, and Mast TD, “Quantitative frequency-domain passive cavitation imaging,” IEEE Trans. Ultrason., Ferroelectr., Freq. Control, vol. 64, no. 1, pp. 177–191, Jan. 2017. [Online]. Available: https://www.ncbi.nlm.nih.gov/pmc/articles/PMC5344809/27992331 10.1109/TUFFC.2016.2620492 PMC5344809

[35] Thies M and Oelze ML, “Real-time visualization of a focused ultrasound beam using ultrasonic backscatter,” IEEE Trans. Ultrason., Ferroelectr., Freq. Control, vol. 68, no. 4, pp. 177–191, Apr. 2021. 10.1109/TUFFC.2020.3035784 PMC8081032 33147143

[36] Czarnota GJ , “Tumor radiation response enhancement by acoustical stimulation of the vasculature,” Proc. Nat. Acad. Sci. USA, vol. 109, no. 30, pp. E2033–E2041, Jul. 2012. [Online]. Available: https://www.pnas.org/content/109/30/E2033 22778441 10.1073/pnas.1200053109 PMC3409730

[37] Sharma D , “Ultrasound microbubble potentiated enhancement of hyperthermia-effect in tumours,” PLoS ONE, vol. 14, no. 12, Dec. 2019, Art. no. e0226475. 10.1371/journal.pone.0226475 PMC6919613 31851698

[38] Sharma D , “Optimization of microbubble enhancement of hyperthermia for cancer therapy in an *in vivo* breast tumour model,” PLoS ONE, vol. 15, no. 8, Aug. 2020, Art. no. e0237372. 10.1371/journal.pone.0237372 PMC7428078 32797049

[39] Eisenbrey JR , “Sensitization of hypoxic tumors to radiation therapy using ultrasound-sensitive oxygen microbubbles,” Int. J. Radiat. Oncol. Biol. Phys, vol. 101, no. 1, pp. 88–96, 2018. 29477294 10.1016/j.ijrobp.2018.01.042 PMC5886808

[40] Sheikov N, McDannold N, Vykhodtseva N, Jolesz F, and Hynynen K, “Cellular mechanisms of the blood-brain barrier opening induced by ultrasound in presence of microbubbles,” Ultrasound Med. Biol, vol. 30, no. 7, pp. 979–989, Jul. 2004. 15313330 10.1016/j.ultrasmedbio.2004.04.010

[41] Hynynen K, McDannold N, Sheikov NA, Jolesz FA, and Vykhodtseva N, “Local and reversible blood-brain barrier disruption by noninvasive focused ultrasound at frequencies suitable for trans-skull sonications,” Neuroimage, vol. 24, pp. 12–20, Jan. 2005. 15588592 10.1016/j.neuroimage.2004.06.046

[42] Konofagou EE, Tung Y-S, Choi J, Deffieux T, Baseri B, and Vlachos F, “Ultrasound-induced blood-brain barrier opening,” Current Pharmaceutical Biotechnol, vol. 13, no. 7, pp. 1332–1345, Jun. 2012. 10.2174/138920112800624364 PMC4038976 22201586

[43] Carpentier A , “Clinical trial of blood-brain barrier disruption by pulsed ultrasound,” Sci. Transl. Med, vol. 8, no. 343, 2016, Art. no. 343re2. 10.1126/scitranslmed.aaf6086 27306666

[44] Lipsman N , “Blood-brain barrier opening in Alzheimer’s disease using MR-guided focused ultrasound,” Nature Commun, vol. 9, no. 1, pp. 1–8, 2018. 30046032 10.1038/s41467-018-04529-6 PMC6060168

[45] Klibanov AL, “Microbubble contrast agents: Targeted ultrasound imaging and ultrasound-assisted drug-delivery applications,” Investigative Radiol, vol. 41, no. 3, pp. 354–362, Mar. 2006. 10.1097/01.rli.0000199292.88189.0f 16481920

[46] Bystritsky A , “A review of low-intensity focused ultrasound pulsation,” Brain Stimul, vol. 4, no. 3, pp. 125–136, Jul. 2011. [Online]. Available: http://www.sciencedirect.com/science/article/pii/S1935861X11000490 21777872 10.1016/j.brs.2011.03.007

[47] Blackmore J, Shrivastava S, Sallet J, Butler CR, and Cleveland RO, “Ultrasound neuromodulation: A review of results, mechanisms and safety,” Ultrasound Med. Biol, vol. 45, no. 7, pp. 1509–1536, Jul. 2019. 31109842 10.1016/j.ultrasmedbio.2018.12.015 PMC6996285

[48] Romano CL, Romano D, and Logoluso N, “Low-intensity pulsed ultrasound for the treatment of bone delayed union or nonunion: A review,” Ultrasound Med. Biol, vol. 35, no. 4, pp. 529–536, Apr. 2009. [Online]. Available: http://www.sciencedirect.com/science/article/pii/S0301562908004468 19097683 10.1016/j.ultrasmedbio.2008.09.029

[49] Ji R, Burgess M, and Konofagou E, “Transcranial blood-brain barrier opening and power cavitation imaging using a diagnostic imaging array,” in Proc. IEEE Int. Ultrason. Symp, Oct. 2019, pp. 2–4.

[50] Thies M and Oelze ML, “Planning and real-time monitoring of low intensity focused ultrasound therapies using a diagnostic imaging array,” in Medical Imaging, Ultrasonic Imaging and Tomography, vol. 11602. Bellingham, WA, USA: SPIE, 2021, Art. no. 116020I. 10.1109/TMI.2021.3140176 PMC9199060 34986094

[51] Szabo TL, Diagnostic Ultrasound Imaging: Inside Out. New York, NY, USA: Academic, 2004.

[52] Li P-C and Li M-L, “Adaptive imaging using the generalized coherence factor,” IEEE Trans. Ultrason., Ferroelectr., Freq. Control, vol. 50, no. 2, pp. 128–141, Feb. 2003. 12625586 10.1109/tuffc.2003.1182117

[53] Mallart R and Fink M, “Adaptive focusing in scattering media through sound-speed inhomogeneities: The van Cittert Zernike approach and focusing criterion,” J. Acoust. Soc. Amer, vol. 96, no. 6, pp. 3721–3732, 1994. [Online]. Available: https://asa.scitation.org/doi/10.1121/1.410562

[54] Hollman KW, Rigby KW, and O’Donnell M, “Coherence factor of speckle from a multi-row probe,” in Proc. IEEE Ultrason. Symp., vol. 2, Oct. 1999, pp. 1257–1260.

[55] Montaldo G, Tanter M, Bercoff J, Benech N, and Fink M, “Coherent plane-wave compounding for very high frame rate ultrasonography and transient elastography,” IEEE Trans. Ultrason., Ferroelectr., Freq. Control, vol. 56, no. 3, pp. 489–506, Mar. 2009. 19411209 10.1109/TUFFC.2009.1067

[56] Jensen JA and Svendsen NB, “Calculation of pressure fields from arbitrarily shaped, apodized, and excited ultrasound transducers,” IEEE Trans. Ultrason., Ferroelectr., Freq. Control, vol. 39, no. 2, pp. 262–267, Feb. 1992. 18263145 10.1109/58.139123

[57] Jensen JA, “Field: A program for simulating ultrasound systems,” in Proc. 10th Nordic Baltic Conf. Biomed. Imag, 1996, vol. 4, no. 1, pp. 351–353.

[58] Gauthier M, King DA, and O’Brien WD Jr., “Evaluation of the temporal stability of definity using double passive cavitation detection,” J. Med. Ultrasound, vol. 32, no. 9, p. 1535, 2013. 10.7863/ultra.32.9.1535 PMC3938207 23980212

[59] Pouliopoulos AN , “Temporal stability of lipid-shelled microbubbles during acoustically-mediated blood-brain barrier opening,” Frontiers Phys, vol. 8, p. 137, May 2020. 10.3389/fphy.2020.00137 PMC7250395 32457896

[60] Zhang Y, Tang N, Huang L, Qiao W, Zhu Q, and Liu Z, “Effect of diagnostic ultrasound and microbubble-enhanced chemotherapy on metastasis of rabbit VX2 tumor,” Med. Phys, vol. 48, no. 7, pp. 3927–3935, Jul. 2021. 33774845 10.1002/mp.14867

[61] Sun T, Samiotaki G, Wang S, Acosta C, Chen CC, and Konofagou EE, “Acoustic cavitation-based monitoring of the reversibility and permeability of ultrasound-induced blood-brain barrier opening,” Phys. Med. Biol, vol. 60, no. 23, p. 9079, 2015. 26562661 10.1088/0031-9155/60/23/9079 PMC4668271

